# Caspase-3 Contributes to ZO-1 and Cl-5 Tight-Junction Disruption in Rapid Anoxic Neurovascular Unit Damage

**DOI:** 10.1371/journal.pone.0016760

**Published:** 2011-02-22

**Authors:** Christoph M. Zehendner, Laura Librizzi, Marco de Curtis, Christoph R. W. Kuhlmann, Heiko J. Luhmann

**Affiliations:** 1 Institute of Physiology and Pathophysiology, University Medical Center of the Johannes Gutenberg-University of Mainz, Mainz, Germany; 2 Unit of Experimental Neurophysiology and Epileptology, Fondazione Istituto Neurologico Carlo Besta, Milano, Italy; University of Texas MD Anderson Cancer Center, United States of America

## Abstract

**Background:**

Tight-junction (TJ) protein degradation is a decisive step in hypoxic blood-brain barrier (BBB) breakdown in stroke. In this study we elucidated the impact of acute cerebral ischemia on TJ protein arrangement and the role of the apoptotic effector protease caspase-3 in this context.

**Methodology/Principal Findings:**

We used an in vitro model of the neurovascular unit and the guinea pig whole brain preparation to analyze with immunohistochemical methods the BBB properties and neurovascular integrity. In both methodological approaches we observed rapid TJ protein disruptions after 30 min of oxygen and glucose deprivation or middle cerebral artery occlusion, which were accompanied by strong caspase-3 activation in brain endothelial cells (BEC). Surprisingly only few DNA-fragmentations were detected with TUNEL stainings in BEC. Z-DEVD-fmk, an irreversible caspase-3 inhibitor, partly blocked TJ disruptions and was protective on trans-endothelial electrical resistance.

**Conclusions/Significance:**

Our data provide evidence that caspase-3 is rapidly activated during acute cerebral ischemia predominantly without triggering DNA-fragmentation in BEC. Further we detected fast TJ protein disruptions which could be partly blocked by caspase-3 inhibition with Z-DEVD-fmk. We suggest that the basis for clinically relevant BBB breakdown in form of TJ disruptions is initiated within minutes during ischemia and that caspase-3 contributes to this process.

## Introduction

In vivo the blood-brain barrier (BBB) together with its neuronal and non-neuronal surrounding represents the neurovascular unit (NVU) [1], which is characterized by a functional interaction between brain endothelial cells (BEC), astrocytes, pericytes, microglia and neurons. BEC effectively separate the brain from cerebral blood flow through a complex and precisely regulated system of tight-junction (TJ) proteins [2]. Ischemic conditions induce a breakdown of the BBB which leads to numerous severe clinical complications like brain edema formation and neuroinflammation [3], [4]. It is well documented that TJ proteins play a pivotal role in hypoxic BBB injury [5], [6]. Therefore a better understanding of TJ pathophysiology in acute cerebral ischemia may improve treatment strategies in stroke.

Numerous studies have demonstrated a neuroprotective effect of the irreversible caspase-3 inhibitor Z-DEVD-fmk under ischemic conditions [7]–[10]. It is further known that proteases (e. g. caspase-3, matrix metallo proteases) are involved in ischemic BEC damage [11]. Clinical datasets showed that elevated serum levels of caspase-3 are an indicator for infarct growth and poor outcome after stroke [12]. A recent clinical trial reports that post-stroke treatment with minocycline, a drug with multiple anti-apoptotic effects, improves patient outcome [13]. It is well documented that minocycline inhibits caspase-1 and caspase-3 expression in vivo [14]. These investigations point to a significant involvement of caspases in hypoxic cell damage in the brain.

We used a recently described model of the NVU [15] to analyze (i) acute ischemic alterations of the NVU by immunohistochemistry of the TJ proteins Zonula occludens-1 (ZO-1) and Claudin-5 (Cl-5), which have a key role in the pathogenesis of ischemic blood-brain barrier breakdown [6], [15]–[17], and (ii) whether caspase-3 is involved in altering TJ protein arrangement. To strengthen the significance of our investigation we also performed immunohistochemical analyzes of ZO-1 after middle cerebral artery occlusion (MCAO) in the guinea pig isolated whole brain, in which the BBB as NVU is completely preserved [18].

We observed a Rapid Anoxic Neurovascular Unit Damage (RANUD) which was partly caused by caspase-3 mediated degradation of the TJ proteins ZO-1 and Cl-5.

In contrast to previous findings [19], [20] the results in our present report indicate that the BBB in our models of the NVU is already impaired after short periods of chemical oxygen and glucose deprivation (OGD)/MCAO at the level of the TJs. Furthermore, we identified caspase-3 to be significantly involved in ZO-1 and Cl-5 disorganisation independently from nuclear fragmentation. Based on our data we propose (i) that ischemic alterations of TJs in the NVU differ significantly from the currently discussed concept and (ii) that caspase-3 may be a target for therapeutic intervention in acute cerebral ischemia to stabilize NVU integrity.

## Materials and Methods

### Cell culture

We used a murine brain endothelial cell line bEnd.3 (bEnd.3, American Type Culture Collection, Manassas, Virginia, USA) for coculture experiments as described previously [15]. bEnd.3 were cultured as recommended by the manufacturer and maintained at 37°C and 5% CO_2_. Media consisted of DMEM Glutamax supplemented with 2% Penicillin/Streptomycin (all from Invitrogen GmbH, Karlsruhe, Germany) and 15% fetal calf serum (Biochrom AG, Berlin, Germany). For coculture procedures BEC were grown until confluence was reached on glass coverslips.

### Neurovascular unit model

We used a recently described murine coculture model consistent of cortical organotypic slice cultures (COSC) and the murine brain endothelial cell line bEnd.3 as documented in detail before [15]. All experiments were conducted in accordance with the national and European (86/609/EEC) laws for the use of animals in research and were approved by the local institutional animal care committee. All efforts were made to minimize the number of animals used and their suffering. A detailed description of the coculture was documented previously [15].

A careful examination of the coculture with light microscopy for intact cell morphology before starting experimental manipulations was obligatory since dislocations of the COSC from bEnd.3 before experimental manipulations occasionally occurred. Only cocultures with firm adhesion of COSC on bEnd.3 coverslips and intact cell shapes were used for experiments.

### Guinea pig whole brain preparation

Experiments were performed on brains of young adult Hartley guinea pigs (150–200 g; Charles River, Italy) isolated following the standard technique described elsewhere [18], [21], [22]. Briefly, after anaesthesia, the brain was carefully isolated and transferred to the incubation chamber. A polyethylene cannula was inserted into the basilar artery to ensure arterial perfusion with a complex saline solution (composition in mmol/L: 126 NaCl, 3 KCl, 1.2 KH_2_PO_4_, 1.3 MgSO_4_, 2.4 CaCl_2_, 26 NaHCO_3_, 15 glucose and 3% dextran MW 70.000) oxygenated with a 95% O_2_ - 5% CO_2_ gas mixture (pH 7.3). The rate of arterial perfusion was 7 ml/min. Experiments were carried out at 32 °C. The experimental protocol was approved by the Ethics Committee of the Fondazione Istituto Neurologico “C.Besta”, in accordance with National and International guidelines on care and use of laboratory animals (protocol number 45–46; 07/11/2003).

### Dyes, chemicals and antibodies

2-deoxy-D-glucose, NaCN, propidium iodide (10 mg/l) and triton X-100 were from Sigma-Aldrich (Steinheim, Germany); antibody directed against the cleaved, active form of caspase-3 was from Signaling Technology (Asp175, Cell Signaling Technology Inc.) [23]; polyclonal anti-ZO-1 (61–7300) and anti-claudin-5 (35–2500) were from Zymed ordered via Invitrogen; polyclonal rabbit anti-gial fibrillary acidic protein (GFAP) Z0334 was from DakoCytomation (Glostrup Denmark); biotinylated goat anti-rabbit antibody was from Vector Laboratories (Burlingame, CA, USA); Cy™3-conjugated AffiniPure goat anti-rat IgG and Cy™2-conjugated AffiniPure goat anti-rabbit IgG were from Dianova (Hamburg, Germany); Streptavidin Alexa 488 and Alexa Fluor 568 goat anti-mouse were from Molecular Probes; Streptavidin Cy3 was from Sigma (S6402, Deisenhofen, Germany); Dylight™594-conjugated goat anti-rabbit igG (from Jackson ImmunoResearch, West Grove, PA, USA); anti-MAP-2 primary antibody (MAP-2 clone AP 20, Bio-Optica, Fremont, CA, USA); monoclonal biotinylated horse anti-mouse IgG (Vector Laboratories Inc., Burlingame, CA, USA); avidin-biotin peroxidase protocol (ABC kit, Vector Laboratories Inc.); 3,3′-diamino-benzidine tetrhaydrochloride (DAB; Sigma, St Louise, MO, USA); normal goat serum (from Jackson ImmunoResearch, ordered via Dianova); Fluoromount was from Southern Biotech (Birmingham, AL, USA); Fluorsave was from Calbiochem (San Diego, CA, USA); TUNEL label mix and TUNEL enzyme were both from Roche (Mannheim, Germany); bovine serum albumin was from Dianova; Z-DEVD-fmk was from Calbiochem.

### Oxygen and glucose deprivation

OGD was induced by replacing culture media of the coculture with HBSS supplemented with 2 mmol/l CaCl2, 1 mmol/l MgCl2, 10 mmol/l 2-deoxy-D-glucose and 5 mmol/l sodium cyanide. Exposure time was 30 min. For immunohistochemistry cocultures undergoing OGD were gently washed in HBSS (37°C) before fixation. The caspase-3 inhibitor Z-DEVD-fmk was incubated for 2 hours at a concentration of 50 µmol/l before OGD. A protective and non-toxic effect of Z-DEVD-fmk at this concentration has been demonstrated previously [24].

### Middle cerebral artery occlusion

The proximal portion of one of the middle cerebral artery (MCA) was carefully isolated from the surrounding dura. A silk thread was slid under the MCA and a loose node was prepared around the vessel. To induce transient ischemia, the ends of the loose silk thread positioned around the MCA were pulled by tweezers and the MCA was occluded. MCAO of 30 min duration was induced 90 min after in vitro placement.

### Immunohistochemistry (IHC)

#### Coculture of the NVU

IHC stainings for ZO-1, Cl-5, Caspase-3, GFAP and CD31 in the coculture model of the NVU were performed as described previously [15]. In case COSC dislocated from coverslips during washing procedures for IHC, COSC were analyzed separately from bend3 monolayers on coverslips for tight-junction integrity in cerebral microvessels.

Co-stainings of activated caspase-3 and Terminal deoxynucleotidyl Transferase Biotin-dUTP Nick End Labeling (TUNEL) were performed as follows: After experimental manipulation probes were fixed in paraformaldehyde (4%) for 30 min at room temperature (RT). Thereafter samples were permeabilzed in 0.1 mol/l tri-natrium citrate (freshly prepared) and 0.1% triton X-100 for 2 min on ice in a multiwell plate. Then probes were washed with phosphate buffer saline (PBS) 0.01 mol/l and TUNEL-reaction mixture (50 µl/coculture) was added. Cocultures were covered with parafilm and placed in a humidified chamber (1 h, 37 °C). This was followed by blocking and permeabilisation with 7% normal goat serum and 0.3% triton X-100 in PBS 0.01 mol/l (2 h, RT). The primary antibody against cleaved caspase-3 (dilution: 1∶200 in 2% bovine serum albumin with 0.05% acid and 0.1% triton X-100 in PBS 0.01 mol/l) was incubated over night at RT. Samples were washed with PBS 0.01 mol/l, then secondary antibodies were incubated for 2 h at RT: biotinylated goat anti-rabbit IgG (H+L) 1∶200 and to enhance TUNEL-signal Cy2-anti-FITC 1∶400 diluted with 2% bovine serum albumin with 0.05% acid was applied. Cells were washed again with PBS 0.01 mol/l, incubated with Streptavidin-Cy3 in PBS 0.01 mol/l (1∶400, 1 h, RT), washed once again and finally embedded in Fluoromount or Fluorsave. Activated caspase-3 and propidium iodide (PI) were co-stained after fixation of the COSC for 30 min in 4% paraformaldehyde followed by a careful wash in 0.01 mol/l PBS. Probes were blocked and permeabilized with 7% normal goat serum and 0.3% triton-X100 in 0.01 mol/l PBS for 2 h at RT. Subsequently primary antibodies (dilution: 1∶200 in 2% bovine serum albumin with 0.05% acid and 0.1% triton X-100 in 0.01 mol/l PBS) against cleaved caspase-3 were incubated overnight at RT. After washing with 0.01 mol/l PBS secondary antibodies (biotinylated goat anti-rabbit IgG (H+L) 1∶100 diluted with 2% bovine serum albumin with 0.05% azide) were incubated for 2 h at RT. Afterwards COSC were washed in 0.01 mol/l PBS and were then incubated with Streptavidin Alexa 488 (in 0.01 mol/l PBS diluted at 1∶400 for 1 h at RT). Samples were washed again with 0.01 mol/l PBS, incubated with PI (1∶400 for 10 min at RT) and embedded in Fluorsave.

#### Guinea pig isolated whole brain

For morphological studies, the brains were perfused at the end of the experiment via the basilary artery with a solution of paraformaldehyde-lysine-periodate (PLP) in 0.1 mol/l phosphate buffer (PB), ph 7.4 for 10 min. Post-fixation was carried out in PLP for 2 h at RT. After fixation, serial coronal sections (50 µm) were cut with a vibratome (VT 1000S Leica Heidelberg, Germany).

Selected free-floating vibratome sections were incubated in 0.01 mol/l PBS containing 1% bovine serum albumin and 0.2% triton X-100. Sections were then incubated overnight at 4 °C with anti-ZO-1 primary antibody (1∶400). Then the slices were rinsed in PBS and were incubated for 2 h at RT in a Dylight™594-conjugated goat anti-rabbit igG (1∶600) and, after repeated rinsing, were mounted in Fluorosave and were examined under a confocal laser scanning microscope.

For microtubule-associated protein (MAP-2) staining, the brains were fixed by immersion in a cold 4% paraformaldehyde solution in 0.1 M PB, pH 7.4. On the next day, the brains were cut in serial coronal sections as described before. Selected free-floating slices were incubated in 0.01 M phosphate buffered saline (PBS) containing 10% normal horse serum (NHS) and 0.2% triton X-100. Sections were then incubated overnight at 4 °C with anti-MAP-2 primary antibody (1∶1000), diluted in 1% NHS in PBS. Then the slices were rinsed in PBS and were incubated for 75 min in monoclonal biotinylated horse anti-mouse IgG (1∶200; Vector Laboratories Inc., Burlingame, CA, USA). The avidin-biotin peroxidase protocol (ABC jìkit, Vector Labs) was applied, using 3.3′-diamino-benzidine tetrhaydrochloride (DAB; Sigma, St Louise, MO, USA) as chromogen. After staining, sections were dehydrated, cleared with xilene and coverslipped with DPX.

### Immunofluorescence confocal microscopy (ICM)

For ICM we used an upright microscope (BX51WI, Olympus; Hamburg, Germany), equipped with a Nipkow spinning disk confocal system (QLC10 Visitech, Sunderland, United Kingdom) and a Krypton/Argon laser (Laser Physics, Cheshire, UK) and a confocal laser scanning microscope (Nikon D-ECLIPSE C1). Fluorescent probes were excited at 488 nm and 568 nm. Images were analysed using Metamorph imaging software (Molecular Devices Corp., Downington, CA, USA).

### Measurements of trans-endothelial electrical resistance (TEER)

TEER was analyzed with electrical cell impedance sensing (ECIS) as described in detail before [15]. bEnd.3 were seeded at a density of approximately 50,000 cells per well in an 8 well gold electrode ECIS assay (0.8 cm^2^ growth area, ibidi in cooperation with Applied BioPhysics, Martinsried, Germany). bEnd.3 media were refreshed after 1 day and COSC were gently transferred into the wells. When impedance measurements reached maximal values (cocultures reached TEER values of 890.9±17.9 OHM×cm^2^), OGD was induced to monitor in vitro ischemia-induced BBB breakdown.

### Quantification of Caspase-3 immunoreactivity

To quantify caspase-3 immunoreactivity we combined PI stainings of brain endothelial cell nuclei with cleaved caspase-3 antibody staining. Three randomly chosen fields of view (RFV, magnification 60×) per probe (n = 3 coculture preparations ->9 RFV per probe) in the bEnd.3 monolayer area were acquired under normoxic and OGD conditions. The cell number of each field was determined by counting PI positive cell nuclei. The RFV were divided into 25 fields and caspase-3 positive fields were subsequently counted. The number of immunoreactive caspase-3 quadrants in the RFV was divided through the cell number of each RFV. As a result the amount of active caspase-3 fields per cell in% was obtained.

### Quantification of tight-junction impairment

Length of TJ disruption was measured (in pixels) and related to the whole length of ZO-1 and Cl-5 staining in corresponding microvessels (modified according to [25]). TJ disruptions in percentage (%) are presented. Only vessels that were immunoreactive for both ZO-1 and Cl-5 were used for quantification. For each condition at least 26 microvessels from 6-7 coculture preparations were analyzed. For quantification of TJ impairment in the brain endothelial monolayer area of the NVU in vitro model, we used Metamorph imaging software similar as documented before [26]. Cells were analyzed with linescan measurements and intensity histograms. Intensity values at the cell membranes (represented by Value 1: V1 and Value 3: V3) were measured and set in relation to the average intensity of the cytoplasm (average value of the distance between V1 and V3 -> Value 2: V2) of the corresponding cell. The following formula was used for calculation of the relative intensity values of ZO-1 and Cl-5: [(V1+V3)/2] /V2.

For each condition 12 arbitrarily chosen cells from 3 coculture preparations were analyzed.

### Statistics

Results are expressed as mean values ± s.e.m. A value of P<0.05 was considered as significant. Statistically significant effects of OGD on differences in TEER values, TJ damage and cleaved caspase-3 levels were assessed by t-test analyses.

## Results

### Expression of ZO-1 and Cl-5 in cortical microvessels of the in vitro neurovascular unit model

**Figure 1:**
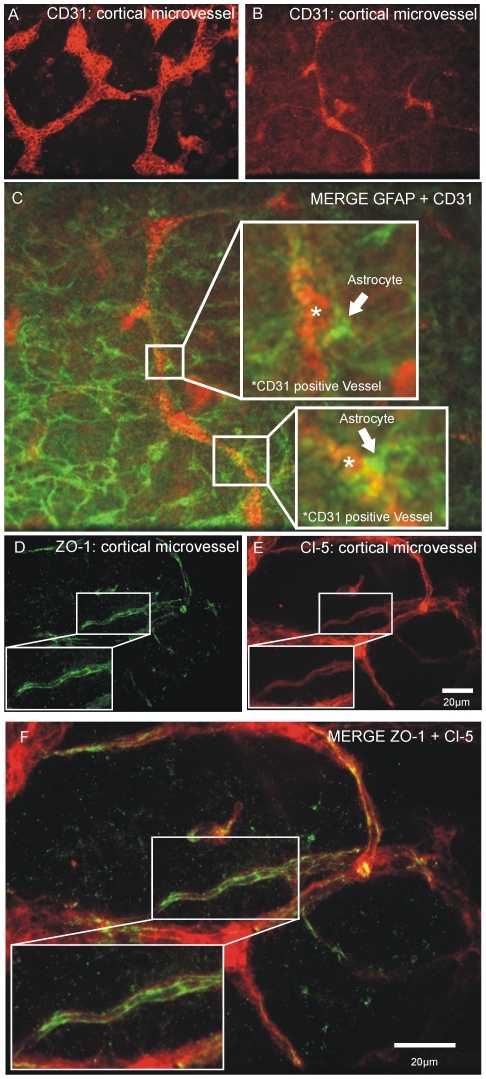
Expression of ZO-1 and Cl-5 in cortical microvessels of the neurovascular unit in vitro model. (A, B) CD31 stainings demonstrating preservation of microvascular morphology in the COSC of the coculture. (C), Endfeet of Astrocytes (arrowheads in C) contacting CD31 positive microvessels. D-F, Microvessels express the tight-junction proteins ZO-1 (panel D) and Claudin-5 (panel E), which show a co-localization (panel F). Scale bars in C and F: 20 µm; in A, B and D, E: 20 µm.

Survival of microvessels in cortical organotypic slice cultures is critical [27]. We found cortical microvessels with an intact morphology in the cortical area of our in vitro NVU model [15]. Immunofluorescence images of CD31 (Fig. 1A, B) show an intact microvascular morphology. We found endfeet of astrocytes in contact with CD31 positive microvessels (Fig. 1C). Antibody staining against the TJ proteins ZO-1 and Cl-5 revealed a prominent expression of these proteins within the cortical microvessels (Fig. 1D–F).

### Alterations of tight-junction protein arrangement after oxygen and glucose deprivation and effects of Z-DEVD-fmk on tight-junction integrity and TEER

**Figure 2:**
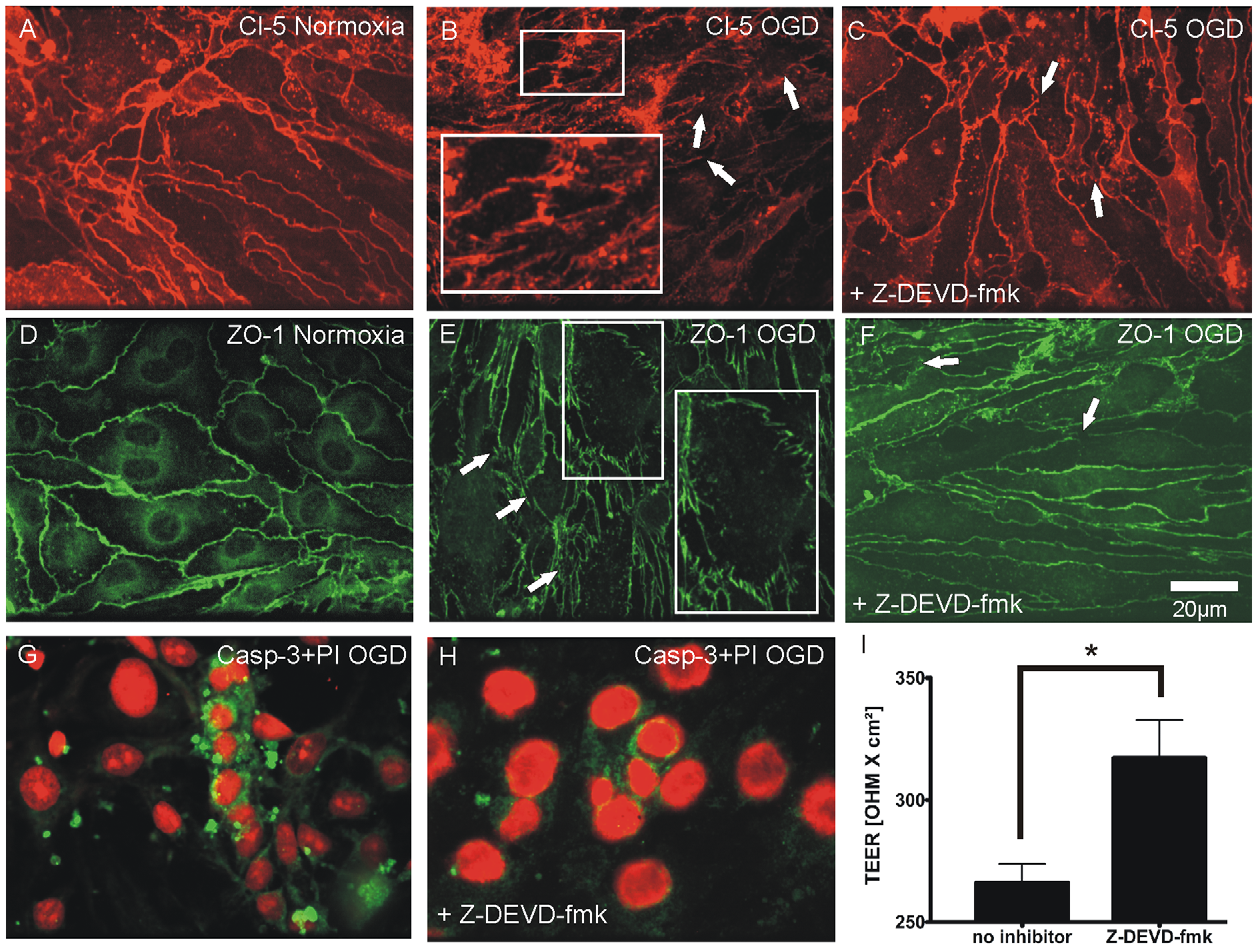
Application of Z-DEVD-fmk preserves TEER and TJ monolayer integrity under OGD. (A-F) Continuous expression of Cl-5 (panel A) and ZO-1 (panel D) under normoxic conditions. After 30 min of OGD Cl-5 and ZO-1 are prominently disrupted (arrowheads and insets in B and E) and display a ragged shape. Analogous to the protective effect of Z-DEVD-fmk on TEER (panel I), Z-DEVD-fmk also has a protective effect on ZO-1 and Cl-5 expression, but does not completely prevent TJ disruption (arrowheads in C and F). (G) Immunohistochemical co-staining with propidium iodide (red) and caspase-3 (green) shows high amounts of cleaved caspase-3 after OGD which are lowered by pre-treatment with Z-DEVD-fmk (H). I, Pre-treatment with 50 µmol/l Z-DEVD-fmk for 2 hours preserves TEER values significantly after hypoxia (no inhibitor 266.1±7.7 OHM×cm^2^ vs. Z-DEVD-fmk: 317.3±15.5 OHM×cm^2^, n = 6 coculture preparations, P<0.05).

Under normoxic conditions a continuous alignment of Cl-5 and ZO-1 could be observed (Fig. 2A, D). OGD for 30 minutes induced a disruption of Cl-5 and ZO-1 in the monolayer area of the NVU model (arrowheads and insets in Fig. 2B, E). In order to address the question whether the activation of caspase-3 contributes to the detected rapid TJ impairment, we applied the caspase-3 inhibitor Z-DEVD-fmk [28]. Z-DEVD-fmk had a protective effect on TJ integrity. Only a few disruptions were visible in probes pre-treated with Z-DEVD-fmk (arrowheads in Fig. 2C, F) after OGD. However, the disruption of ZO-1 and Cl-5 was not completely abolished. Furthermore, Z-DEVD-fmk partly reduced caspase-3 activation (Fig. 2G, H). Because trans-endothelial electrical resistance (TEER) correlates with blood-brain barrier integrity [19] we also performed TEER measurements and observed that pre-treatment with Z-DEVD-fmk preserved NVU integrity. TEER values of probes treated with Z-DEVD-fmk had significantly higher TEER values after OGD compared with probes that were not treated with the inhibitor (Fig. 2I, Z-DEVD-fmk: 317.3±15.5 OHM×cm^2^ vs. no inhibitor 266.1±7.7 OHM×cm^2^, P<0.05, n = 6 coculture preparations), which correlates well with our immunohistological findings (Fig. 2B, C, E and F). To quantify the observed TJ damage in the monolayer area of our NVU in vitro model we analyzed the localization of ZO-1 and Cl-5 in BEC with linescan measurements similar as described previously by Brown and colleagues [26]. It is known that BBB breakdown correlates with a redistribution of TJ from the plasma membrane to the cytoplasm which goes along with TJ disruptions [29]. To quantify subcellular TJ redistributions the intensity of ZO-1 and Cl-5 in BEC was measured with linescans. Intensity values at cell borders were set in relation to the average intensity of the cytoplasm.

**Figure 3:**
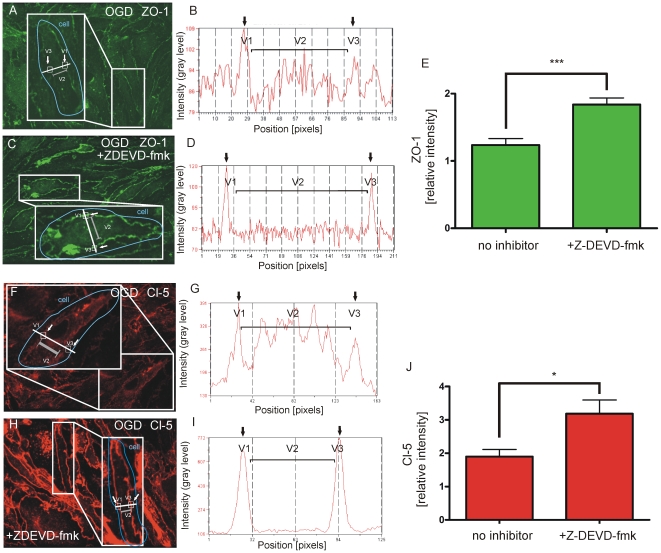
Z-DEVD-fmk preserves TJ expression in brain endothelial cell membranes under OGD. Fluorescence intensity of ZO-1 and Cl-5 in cell membranes related to the fluorescence intensity of the cytoplasm: Cell membranes are represented by values in V1 and V3. The fluorescence intensity of the cytoplasm is the average intensity of the distance V1-V3: V2. (A-E) Intensity histograms of ZO-1 in cells that were not pre-treated with Z-DEVD-fmk before OGD (A and B) have lower peak values at cell membranes (B) related to the intensity of the cytoplasm of the corresponding cell. (C and D) In contrast, high peak values in relation to the intensity of the cytoplasm were observed in cells that were pre-treated with Z-DEVD-fmk. (E) Z-DEVD-fmk significantly preserved ZO-1 intensity at cell membranes under OGD (no inhibitor: 1.24±0.1 vs. Z-DEVD-fmk: 1.84±0.1, n = 12 cells from 3 coculture preparations, P<0.001). (F–J) Quantification of Cl-5 at cell membranes demonstrates that Z-DEVD-fmk has a protective effect on Cl-5 alignment at cell membranes. (F) BEC with impaired Cl-5 expression at the cell membrane as shown in the corresponding linescan histogram (G). (H) Preserved Cl-5 localization at the cell membrane in a BEC pre-treated with Z-DEVD-fmk before OGD with sustained peak values in the linescan histogram (I). (J) Quantification of linescan histograms shows a significant protective effect of Z-DEVD-fmk for Cl-5 expression at cell membranes (no inhibitor: 1.9±0.21 vs. Z-DEVD-fmk: 3.19±0.41, n = 12 cells from 3 coculture preparations, P<0.05).

Cells that were not pre-treated with Z-DEVD-fmk (Fig. 3A, B) had reduced intensity values of relative ZO-1 staining at cell membranes. In contrast, pre-treatment with Z-DEVD-fmk preserved intact ZO-1 localization (Fig. 3C, D) as demonstrated by clear peak values of ZO-1 at cell borders in linescan histograms. Localization of ZO-1 in cell membranes was significantly higher if cells were pre-treated with Z-DEVD-fmk (Fig. 3E, no inhibitor: 1.24±0.1 vs. Z-DEVD-fmk: 1.84±0.1, n = 12 cells from 3 coculture preparations, P<0.001). Similar results were obtained for Cl-5. Z-DEVD-fmk also had a protective effect on Cl-5 under OGD. OGD caused a loss of peak values in BEC cell membranes (Fig. 3F and G). Z-DEVD-fmk sustained clear peak values of Cl-5 staining at the cell membrane (Fi. 3 H, I and J: no inhibitor: 1.9±0.21 vs. Z-DEVD-fmk: 3.19±0.41, n = 12 cells from 3 coculture preparations, P<0.05).

**Figure 4:**
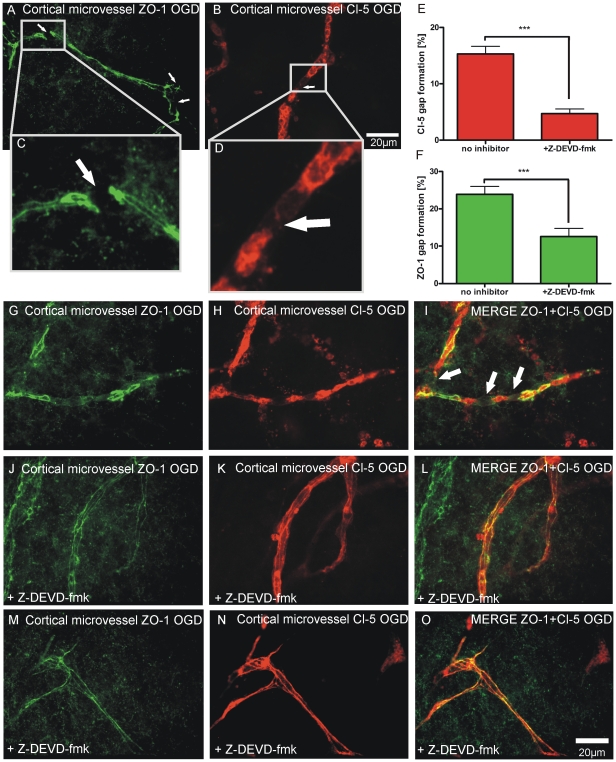
OGD induced microvascular TJ disruption is prevented by Z-DEVD-fmk. (A-D), Depletion of oxygen and glucose results in disruptions of ZO-1 (arrowheads in A, C) and Cl-5 (arrowheads in B, D, I) in cortical microvessels in the NVU model. (E and F) Z-DEVD-fmk significantly reduced gap formation in Cl-5 (15.33±1.32% no inhibitor vs. 4.69±0.84% +Z-DEVD-fmk, P<0.001, n = 28–46 microvessels, 6–7 coculture preparations) and ZO-1 (23.87±2.15% no inhibitor vs. 12.59±2.15% +Z-DEVD-fmk, P<0.001, n = 26–42 microvessels, 6–7 coculture preparations) after 30 min of OGD. (J-O) Here, Z-DEVD-fmk prevents disruptions of ZO-1 and Cl-5.

Analogous to the TJ damage in the BEC monolayer area of the NVU model, OGD resulted in prominent disruptions of ZO-1 and Cl-5 in cortical microvessels (arrowheads Fig. 4A–D; G–I). Quantification of TJ disruptions in cortical microvessels revealed that Z-DEVD-fmk significantly reduced the amount of gap formations (Fig. 4J–O) in both junctional proteins Cl-5 (Fig. 4E, 15.33±1.32% no inhibitor vs. 4.69±0.84% +Z-DEVD-fmk, P<0.001, n = 28-46 microvessels, 6–7 coculture preparations) and ZO-1 (Fig. 4F, 23.87±2.15% no inhibitor vs. 12.59±2.15% +Z-DEVD-fmk, P<0.001, n = 26-42 microvessels, 6–7 coculture preparations).

### Evaluation of ZO-1 and MAP-2 immunohistochemistry in the guinea pig isolated whole brain after MCAO

**Figure 5:**
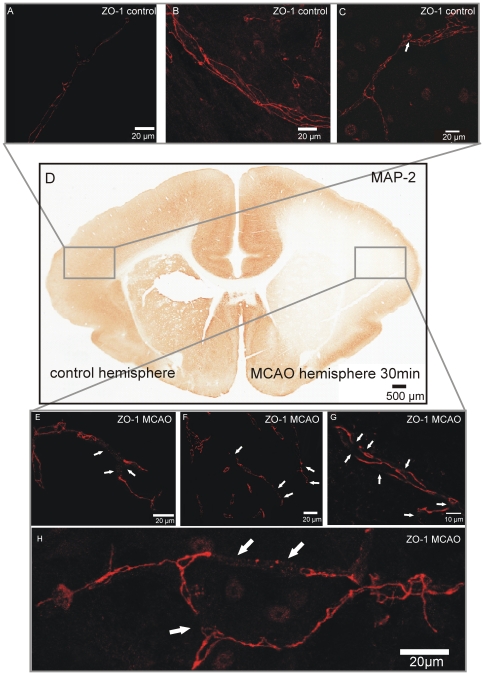
ZO-1 disruption and MAP-2 damage in the guinea pig isolated whole brain after MCAO. (A-C) Cerebral microvessels have continuous ZO-1 alignment in the control hemisphere, with only slight discontinuities in ZO-1 staining (arrowhead in C). (D) MAP-2 staining demonstrates early hypoxic tissue damage after 30 min of MCAO without reperfusion in the occluded MCA-territory of the guinea pig whole brain. The control hemisphere shows no hypoxic damage. (E-H) Numerous disruptions of ZO-1 in cortical microvessels were found after 30 min of MCAO without reperfusion (arrowheads in E-H).

Morphological brain tissue evaluation performed at the end of 30 min of MCAO confirmed the presence of post-ischemic changes in brains either in ZO-1 and MAP-2 staining. ZO-1 staining showed a preservation of the endothelial junctional molecule ZO-1 in the control hemisphere (Fig. 5A–C). MAP-2 is a sensitive marker for early hypoxic brain tissue damage [30], [31]. MAP-2 immunoreactivity was preserved in the unaffected control hemisphere (Fig. 5D), as shown by the homogeneous staining across cortical regions whereas the MAP-2 signal was markedly reduced in brain areas supplied by the occluded MCA (Fig. 5D). Here, a strong impairment of ZO-1 alignment is present in the ischemic hemisphere, as demonstrated by the numerous discontinuities in cortical vessels (Fig. 5E–H).

### Caspase-3 activation and TUNEL staining after 30 min of oxygen and glucose deprivation

**Figure 6:**
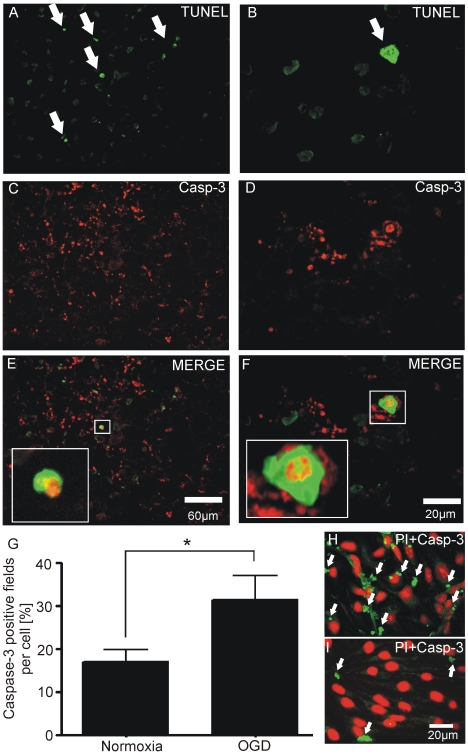
OGD induced caspase-3 expression is only partially associated with DNA-fragmentation. (A, B) Positive TUNEL-labeling demonstrates DNA-fragmentation after 30 min of OGD in the bEnd.3 cells (arrowheads in A and B). (C-F) Elevated levels of active caspase-3 (C and D: red stained immunoreactive cleaved caspase-3) are only partially expressed in TUNEL positive cells (insets in E and F, note the fragmentation of the nucleus in F). (G), Caspase-3 immunoreactivity is significantly higher (arrowheads H) after 30 min of OGD as compared to normoxic (arrowheads I) conditions in the brain endothelial monolayer area of the neurovascular unit model (normoxia: 16.85%±2.78% caspase-3 positive quadrants per cell vs. OGD: 31.31%±5.71% caspase-3 positive quadrants per cell, n = 3 coculture preparations: 9 RFV per group, P<0.05).

Caspase-3 is mainly regarded as a major apoptosis effector protease and was found to be involved in brain endothelial cytotoxicity in hypoxia followed by reoxygenation [11]. Data from patients with high serum levels of caspase-3 point to a poor outcome after stroke compared to patients with low serum levels of caspase-3 [12]. Therefore we were interested in the question whether caspase-3 also has a significant role in fast responses of the NVU in acute cerebral ischemia. We performed double stainings of caspase-3 and TUNEL to analyze whether the activation of caspase-3 is related with DNA-fragmentation, a hallmark for apoptosis [32]. Surprisingly we observed that only a few cells with activated caspase-3 were localized in TUNEL-positive cells (Fig. 6A–F). Levels of activated caspase-3 were higher after 30 min of OGD compared with normoxic conditions (Fig. 6G-I). Values were 16.85±2.78% caspase-3 positive fields per cell (normoxia) vs. 31.31±5.71% caspase-3 positive fields per cell (hypoxia, n = 3 coculture preparations, P<0.05; Fig. 6G).

## Discussion

The main findings of our investigation are: i) The impairment of the TJ proteins already occurs after 30 min OGD (ZO-1 and Cl-5) and MCAO (ZO-1), we refer to this observation as Rapid Anoxic Neurovascular Unit Damage (RANUD); ii) caspase-3 is strongly activated during 30 min of OGD, contributes to TJ disorganisation and is only partly associated with DNA-fragmentation; iii) Z-DEVD-fmk has a protective effect on TJ protein arrangement and TEER in OGD.

Previous investigations have documented that disorganisation of TJ proteins are likely to occur within hours to days after ischemia, especially in models of ischemia followed by reperfusion [3], [6], [16], [20], [33], [34]. Studies that were performed in vitro have mostly used models that lack a preserved neuronal compartment [5], [11], [35]. However, based on previous findings [36] we propose that the close interactions between all cell types of the NVU need to be considered in the pathophysiology of cerebrovascular diseases. For example it has been demonstrated that glia is of major importance for BBB injury in hypoxia [20], [37]. Furthermore, it is known that hypoxia leads to cortical spreading depression in neuronal circuits, which significantly disrupts BBB integrity [38].

In this investigation we used models of the BBB, which imply a preserved neuronal compartment with all cell types of the NVU [15], [18]. We postulate that the results of our in vitro approach are therefore comparable to the in vivo situation. To our knowledge our data demonstrate for the first time that the apoptosis protease caspase-3 is rapidly activated and contributes to tight-junction impairment in RANUD within minutes after onset of hypoxia. Since the immediate depletion of oxygen and glucose metabolism represents a very strong anoxic cell stress, we propose that this in vitro OGD condition is mostly comparable to the ischemic core in stroke in vivo. However, we also detected disruptions of ZO-1 after MCAO in the cortical MCA territory of the guinea pig whole brain preparation which resembles more closely the in vivo situation. Although our experimental in vitro conditions do not entirely reflect the in vivo situation during stroke (e.g. lack of blood components). We propose that the phenomenon of RANUD is very likely to be found in vivo after stroke because in good agreement with our data Gerriets and colleagues [39] detected a vasogenic edema formation with MR-imaging and Evan's Blue extravasation already after 20–45 min of permanent MCAO in an in vivo rodent model without reperfusion. The results of Gerriets and colleagues may be explained by RANUD in the ischemic core region as demonstrated in the present report. However, the protective effect of Z-DEVD-fmk and caspase-3 activation in our study was observed in vitro.

The caspase-3 inhibitor Z-DEVD-fmk had a beneficial effect on TEER and TJ alignment in BEC monolayers and cortical microvessels. These findings are in good agreement with data from Bojarski and colleagues [40], who previously documented a protective effect on ZO-1 alignment in apoptotic Madine-Darby canine kidney cells by inhibiting caspase-3 activity with Z-DEVD-fmk. In our study Z-DEVD-fmk did not fully prevent ZO-1 or Cl-5 disruptions and the loss of TEER after 30 min of OGD. The protective effect of Z-DEVD-fmk in vitro on TEER was partial and is in concordance with the immunohistological findings of ZO-1 and Cl-5 integrity, which were also not completely protected by Z-DEVD-fmk after OGD. This result can be explained by the fact, that other pathophysiological mechanisms contribute to hypoxic BBB injury, like the formation of reactive oxygen species [41], matrix metallo proteases [6], cytokines [42], excessive levels of glutamate [15], [41], phosphorylation of the myosin light chain kinase [43], vascular endothelial growth factor [35], and cortical spreading depression [38].

Our interpretion of a pivotal role of action of caspase-3 in ischemic conditions is also supported by recent clinical datasets. It has been shown that heightened serum levels of caspase-3 correlate with poor outcome after stroke [12] and that minocycline, which is known to inhibit caspase-3 expression [14] in vivo, has a beneficial effect for patient outcome when administrated after stroke [13]. In our study the amount of caspase-3 and TUNEL-positive cells differed significantly. TUNEL-positive cells, which indicate DNA-fragmentation [44], and the detection of active caspase-3 are regarded as a good parameter for apoptosis [45]. Double stainings showed more active caspase-3 than TUNEL-positive BEC after 30 min of OGD. Superimposed images of caspase-3 and TUNEL-labeling revealed that active caspase-3 was only partly associated with DNA-fragmentation.

We suggest that after 30 min OGD caspase-3 is rapidly activated and alters ZO-1 and Cl-5 integrity, but does not lead to DNA-fragmentation and apoptosis at the time point of 30 min anoxia. An alternative explanation for the discrepancy between TUNEL and caspase-3 staining could be an activation of caspase-3 without cell death. It has been previously demonstrated that hypoxia induced activation of caspase-3 does not necessarily induce irreversible apoptotic cell death in neurons [46]. Moreover, it has been recently also documented that caspase-3 plays a major role in Alzheimer's disease that is not instantaneously associated with cell death [47]. These findings demonstrate that caspase-3 may also play a central role in neurodegenerative diseases independently from apoptosis. However, active caspase-3 clearly leads to apoptosis if the duration of hypoxia is expanded: Lee and Lo [11] documented in a human brain endothelial cell model in vitro that longer periods of hypoxia (4 hours) followed by reoxygenation induce an activation of caspase-3 by matrix metallo proteases causing apoptosis and DNA-fragmentation. Further investigations are required to clarify the molecular pathways involved in caspase-3 cleavage during RANUD. The observed TJ alterations in our study could be caused by degradation of ZO-1 and Cl-5 via activated caspase-3. It has been shown that ZO-1 is a cleavage substrate of caspase-3 [40] under apoptotic conditions. It will be interesting to investigate whether this mechanism takes place in our experimental setting and if Cl-5 is also a cleavage substrate of caspase-3. Another important factor could be the cytoskeleton protein actin. The cytoplasmic ZO-1 links actin filaments and the transmembrane protein Cl-5 [1]. An impairment of the actin filaments may therefore also lead to a dislocation of ZO-1 and Cl-5. To evaluate these hypotheses further experimental studies that deal with the molecular effector mechanism of caspase-3 in RANUD within the neurovascular unit are required.

The rapid impairment of tight-junction proteins within minutes after the onset of ischemia may not only be crucial for the development of a vasogenic edema, but may also contribute to the infiltration of inflammatory T-lymphocytes into the brain, as it has been shown to be of decisive importance for delayed post-ischemic brain inflammation [4]. Further in vivo investigations are required to address this question.

In conclusion, our study demonstrates that tight-junction disruption in the neurovascular unit may occur much earlier than previously documented and does not depend on reoxygenation. In addition we show that caspase-3 essentially contributes to ZO-1 and Cl-5 disruption in the early phase of ischemia while only sparse DNA-fragmentation is detectable. Our results may be of importance for the treatment and understanding of the pathogenesis of cerebral ischemia and delayed brain inflammation within the neurovascular unit.
